# GeneExt: a gene model extension tool for enhanced single-cell RNA-seq analysis

**DOI:** 10.1093/bioinformatics/btag094

**Published:** 2026-03-02

**Authors:** Grygoriy Zolotarov, Xavier Grau-Bové, Arnau Sebé-Pedrós

**Affiliations:** Centre for Genomic Regulation (CRG), Barcelona Institute of Science and Technology (BIST), Barcelona, Spain; Universitat Pompeu Fabra (UPF), Barcelona, Spain; Centre for Genomic Regulation (CRG), Barcelona Institute of Science and Technology (BIST), Barcelona, Spain; Centre for Genomic Regulation (CRG), Barcelona Institute of Science and Technology (BIST), Barcelona, Spain; Universitat Pompeu Fabra (UPF), Barcelona, Spain; ICREA, Barcelona, Spain; Tree of Life Program, Wellcome Sanger Institute, Hinxton, UK

## Abstract

**Motivation:**

Incomplete gene models negatively impact single-cell gene expression quantification. This is particularly true in non-model species where often gene 3ʹ ends are inaccurately annotated, while most scRNA-seq methods only capture the 3ʹ transcript region. This results in many genes being incorrectly quantified or not detected.

**Results:**

GeneExt leverages scRNA-seq data to refine gene annotations. We exemplify GeneExt usage and its impact on the gene expression quantification of eight non-model organism single-cell atlases. By extending and homogenizing gene annotations, our tool will help improve biological interpretation and cross-species comparisons of cell type expression atlases.

**Availability:**

GeneExt is available at https://github.com/sebepedroslab/GeneExt (DOI: https://doi.org/10.5281/zenodo.18712940) under a GNU General Public license, together with test data and usage instructions.

## 1 Introduction

Single-cell transcriptomics has transformed the study of cell type diversity across organisms. This technology enables the large-scale and minimally biased molecular characterization of cell types at the whole-organism level, opening the window to cross-species comparisons, discovery of novel cell types, and understanding of gene regulatory programs (Tanay and Sebé-Pedrós 2021). An important problem that hampers the analysis and interpretation of scRNA-seq data in non-model species is the inaccuracy of gene annotations (missing genes, partial genes, etc.) (Mudge and Harrow 2016, Amaral *et al.* 2023, Guigó 2023). The problem is aggravated by the fact that most scRNA-seq methods profile the 3ʹ end of the transcript, where UTRs are often particularly difficult to annotate (Legnini *et al.* 2019, Wang *et al.* 2021, Zolotarov *et al.* 2022, Haese-Hill *et al.* 2023). Thus, a large fraction of sequencing reads map to non-genic regions of the genome and many genes are missing from single-cell expression matrices. This affects both downstream analysis (e.g. cell clustering) and the biological interpretation of the single-cell atlases [e.g. the possibility to miss-quantify key marker genes, or to randomly miss orthologs in cross-species comparisons (Weisman, Murray and Eddy 2022)]. Here, we introduce GeneExt, a tool that addresses 3ʹ end annotation and other related gene annotation problems typically associated with non-model organism single-cell RNA-seq data analysis.

## 2 Methods

### 2.1 GTF/GFF pre-processing

Genome annotation is usually represented by a tabular file where each row corresponds to a single genomic feature. The hierarchical relationships between features are stored in the 9th column of the file. Thus, any re-ordering of the file creates problems for the downstream tools that aim to infer such relationships. Another relatively common problem is missing unique IDs for the features. In the case of one-transcript-per-gene annotations, the transcripts are often assigned the same IDs as their parent genes which makes these IDs non-unique. GeneExt attempts to solve some of these problems by the following:

1. It uses gffutils to parse the hierarchical relationships between the features
2. It adds missing “gene” features
3. It only outputs relevant attributes such as “ID” and “Parent,” in case of GFF or “gene_id”/“transcript_id” in case of GTF

In addition, GeneExt can be used to resolve overlaps between genes on the same strand by:

1. Removing the genes fully contained within another gene
2. Giving priority to the upstream gene

Giving priority to an upstream gene is motivated by the 3ʹ bias in the single-cell RNA-seq data. A signal is more likely to come from a 3ʹ-UTR of an upstream gene than from the 5ʹ-UTR of the downstream gene.

### 2.2 BAM file processing and peak calling and filtering

If requested, the alignment file is subsampled (*--subsamplebam*). The reads are then split by strand, and peaks are called in each strand using MACS2 software (Zhang *et al.* 2008) using the following parameters:

*macs2 callpeak -t tmp/minus.bam -f BAM --keep-dup 20 -q 0.01 --shift 1 --extsize 100 --broad --nomodel --min-length 30 -n minus*

### 2.3 Gene extension and peak clustering

Called peaks are filtered based on the scRNA-seq coverage, calculated per peak as the total coverage divided by the length of the peak. Then, the coverage of the intergenic peaks is compared to the coverage of the peaks falling within genic regions. Intergenic peaks with normalized coverage exceeding the n-th percentile of genic peak coverage (*--peak_perc*; 25th percentile by default) are then kept for extension.

### 2.4 Reanalysis of non-model organism single-cell atlases

For each of the species, three versions of the gene annotations were generated:

1. Original annotation refined by GeneExt (*--clip_5prime*)
2. Output of GeneExt (*--clip_5prime*, *-m* 5000,*--subsamplebam* 100000000)
3. Output of GeneExt (*--clip_5prime*, *-m* 5000, *--subsamplebam* 100000000, *--orphan*)

That is: subsample the dataset to 100M reads; extend the genes to a maximum of 5000 bp downstream; clip 5ʹ overlaps in the genome annotation; use 25th coverage percentile for peak filtering; and keep orphan peaks.

For single-cell atlases obtained with the MARS-seq scRNA-seq technology (*A. queenslandica, M. leidyi, T. adhaerens, N. vectensis*, and *S. pistillata*), we first mapped reads onto the corresponding genome using STAR 2.7.3 (Dobin *et al.* 2013), with parameters: *--outFilterMultimapNmax* 20 --*outFilterMismatchNmax* 8 --*alignIntronMax* 3500. Then we quantified gene expression for each of the three interval sets described above using the MARS-seq pipeline as previously described (Keren-Shaul *et al.* 2019). For *Xenia* sp. we only used the tentacle dataset (10x Chromium v2), and for *O. vulgaris* we only used the single-cell RNA-seq dataset (10x Chromium v3), not single-nuclei data. The R2 reads from each dataset were aligned using STAR v2.7.10a (Dobin *et al.* 2013) to the corresponding genomes (Hu *et al.* 2020, Destanović *et al.* 2023). The resulting alignment files were used as an input for GeneExt with the parameters specified above. The obtained genome annotations were used as an input to STARsolo (Kaminow *et al.* 2021) with default parameters *(*--*soloCBlen* 16 --*soloUMIlen* 12 was used in the case of *O. vulgaris* to account for v3 Chromium chemistry).

### 2.5 Benchmarking in model species by 3ʹ UTR truncation

Genome annotations for *D. melanogaster* (Ensembl Metazoa 49, BDGP6.28), *C. elegans* (Ensembl Metazoa 49, WBcel235), and *M. musculus* (Mmus Ensembl 102) were processed to remove non-protein coding genes and to retain the longest isoform per gene. The resulting annotations (we refer to them as “original” annotations) were modified by removing 3ʹ UTRs, resulting in “truncated” annotations. Publicly available scRNA-seq data for *D. melanogaster* (Li *et al.* 2022), *C. elegans* (Smith *et al.* 2024), and *M. musculus* (Gupta *et al.* 2022) was subsampled to 100 million reads and mapped to the corresponding genome using STAR v2.7.10a (Dobin *et al.* 2013).

Next, each input genome and alignment file were processed using GeneExt and peaks2utr with default parameters, allowing a maximum extension corresponding to the median transcript length (2406 for *M. musculus*, 1623 for *D. melanogaster*, and 1088 for *C. elegans*). The extended and truncated annotations were compared with the original gene annotations at two levels: extension length (Fig. S1a) and measured expression levels per cell type (Fig. S1b). For length comparisons, the genomic coordinates of all 3ʹ ends from the extended annotations were extracted and compared to those in the original annotation. For gene expression comparisons, the four genomic annotations per species (original, truncated, GeneExt extended, and peaks2utr extended) were used as gene models to quantify single-cell expression using STARsolo (Kaminow *et al.* 2021). We then calculated for each cell cluster the Pearson correlation coefficient between gene expression levels obtained with the original annotation and gene expression levels obtained with each of the modified gene annotations (truncated, GeneExt extended, and peaks2utr extender) (Fig. S1b).

## 3 Results

**Figure 1:**
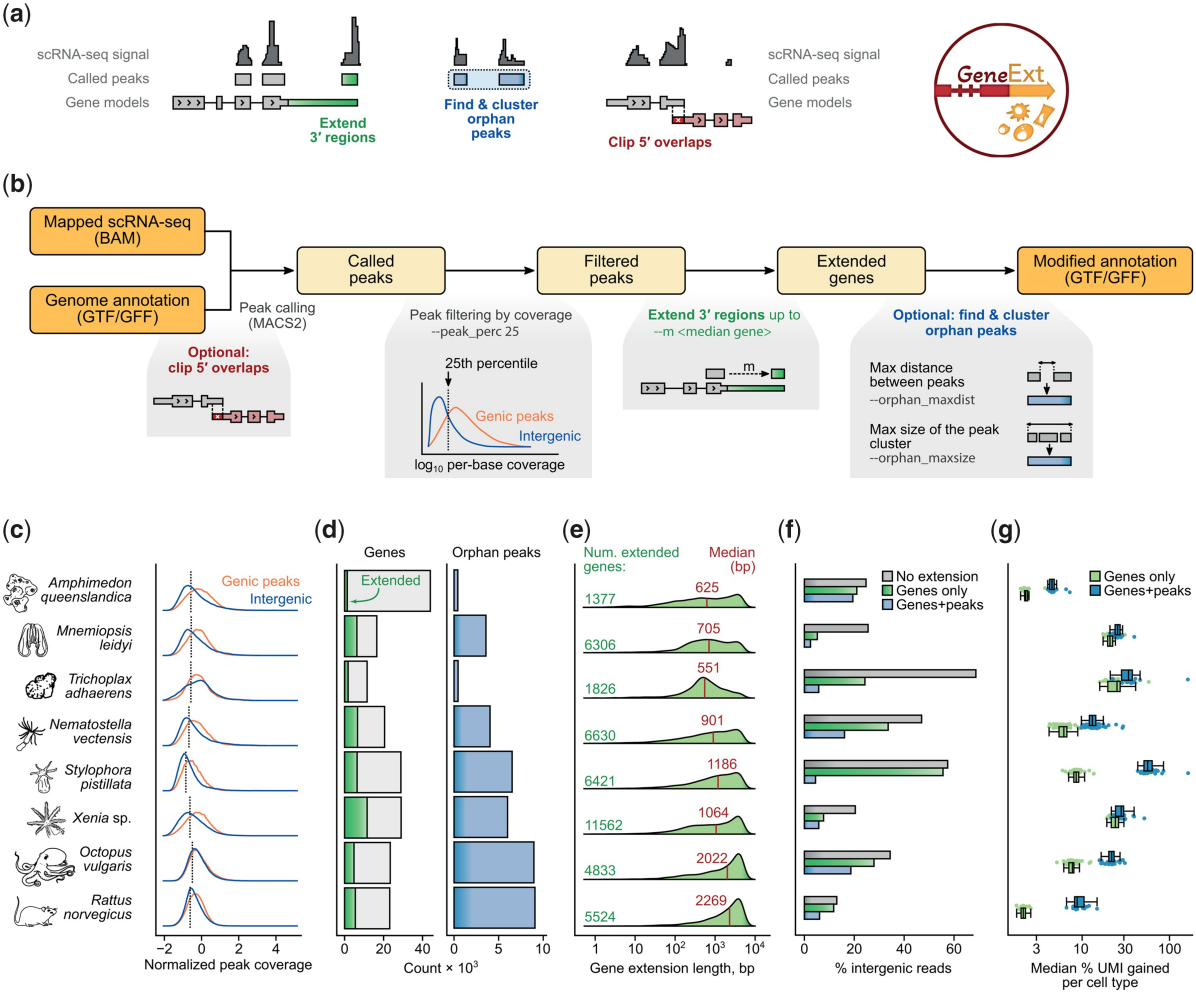
GeneExt tool overview and application to non-model organism cell atlases. (a) GeneExt main function. (b) Schematic representation of the main steps and associated options. (c) Read coverage in called peaks intersecting known exons (“genic peaks”) and intergenic regions. The dashed line indicates the default 25th percentile of genic peaks coverage distribution used to filter intergenic peaks. (d) Barplots indicating the number of extended genes and number of orphan peaks called in different species using GeneExt default parameters. (e) Distributions of gene extension lengths across species. The number of extended genes and the median extension length are indicated. (f) Barplots indicating the percentage of scRNA-seq reads mapping to intergenic regions before and after extending gene annotations in each species. (g) Boxplots indicating median single-cell UMI gains after extending gene annotations and stratified by cell types (individual dots). Gains are calculated as a median percentage of increase relative to the original UMI counts in each cell.

GeneExt takes as input scRNA-seq mapped reads and a gene annotation file (GTF or GFF, any version) and outputs an extended gene annotation file ready for scRNA-seq analysis. The main functions of GeneExt are (Fig. 1a and b):

1. Extension of 3ʹ regions of known genes to better capture reads from 3ʹ-biased scRNA-seq technologies. This is the core functionality of GeneExt and is performed by default. First, GeneExt will define transcriptionally active regions using a user-provided BAM alignment file (e.g. the one produced by CellRanger or other tools) and MACS2 (Zhang *et al.* 2008) to identify stranded peaks, which are classified as genic (mapping to previously annotated gene regions) and intergenic. Intergenic peaks are used to extend the 3′ regions of nearby genes in the same strand, up to a certain distance (*-m* flag). Spurious peaks are removed by excluding those with low coverage (by default, intergenic peaks with coverage below the 25th percentile of the genic peak coverage distribution are excluded; *--peak_perc* flag).
2. Identification of orphan peaks that could constitute unannotated genes or longer 3ʹ UTRs. This is enabled with the --*orphan* flag. Here, GeneExt uses the intergenic peaks not previously assigned to an upstream gene (and passing the coverage filter threshold) to add new features to the final annotation. GeneExt attempts to merge these peaks into clusters defined by a maximum distance between the peaks (--*orphan_maxdist* parameter, 75th percentile of intron length distribution by default) and filtering out peak clusters above a maximum size (*--orphan_maxsize* parameter, median gene length by default). The idea is to avoid peaks representing exons from the same unannotated gene contributing independently to the final UMI count matrix. Clustering of orphan peaks can be disabled with the --*nomerge* flag.
3. Clipping of 5ʹ regions in cases where they overlap with the 3ʹ regions of nearby genes. Depending on the behavior of the UMI demultiplexing software used, this overlap can cause (i) the upstream gene not to be quantified (if 3′ biased scRNA-seq reads mapped into the overlapping region are discarded) or (ii) the downstream gene to have two distinct confounding expression signals (if reads are assigned to both). This optional clipping procedure resolves this ambiguity and is enabled by the --*clip_5prime* flag.
4. In addition, GeneExt will also fix non-standard GTF/GFF files provided by the user (e. g., adding gene features if needed), so as to produce outputs compatible with commonly used UMI demultiplexing software [e.g. 10X CellRanger, STARsolo (Kaminow, Yunusov and Dobin 2021)]. By default, it will only report the longest isoform of each gene, extended according to the options provided.

We tested the effects of these gene annotation modifications using published whole-organism scRNA-seq atlases from diverse species: the sponge *Amphimedon queenslandica*, the ctenophore *Mnemiopsis leidyi*, the placozoan *Trichoplax adhaerens* (Sebé-Pedrós *et al.* 2018a), the cnidarians *Nematostella vectensis* (Sebé-Pedrós *et al.* 2018b), *Stylophora pistillata* (Levy *et al.* 2021) and *Xenia* sp. (Hu *et al.* 2020), the cephalopod *Octopus vulgaris* (in this case only neural tissues) (Styfhals *et al.* 2022), and the rat *Rattus norvegicus* (nucleus accumbens cells) (Savell *et al.* 2020). Together, they represent not only divergent animal lineages, but also diverse scRNA-seq technologies and different genome assembly and annotation qualities. For each species, we quantified single-cell gene UMI counts for three sets of gene annotations: (i) the original annotation, (ii) GeneExt-extended gene models, and (iii) extended gene models plus orphan peaks. In all cases we used default GeneExt parameters, e.g. to filter out intergenic peaks based on genic peak coverage distributions (Fig. 1c).

The fraction of extended genes (Fig. 1d) varies greatly across species, from 3.2% in *A. queenslandica* to 39.9% in *Xenia sp.*, while the number of remaining intergenic orphan peaks goes from 416 in *A. queenslandica* to 9079 in *R. norvegicus*. Similar differences can be found in the length of gene extension (Fig. 1e). This reflects the fact that incomplete or inaccurate gene models, gene over-annotation and/or under-annotation will affect each species differently. For example, the high fraction of modified genes in *Xenia sp.* probably reflects the systematic mis-annotation of 3ʹ UTRs in this genome. The different sources of annotation biases are also reflected by observed differences in intergenic mapping reduction after extension (Fig. 1f). The high reduction in *T. adhaerens* and *M. leidyi* suggests that most missing information came from 3ʹ-incomplete gene models rather than missing genes. In *S. pistillata*, on the other hand, we observed only a small reduction of intergenic reads after extension, which could be explained by a substantial number of missing genes.

We then tested the gain in UMI counts per cell for each cell type (Fig. 1g). As expected, the inclusion of orphan peaks results in higher UMI gains than simply performing 3ʹ gene extension. These effects are highly species-specific (again, related to the varying quality of gene annotations in different species), but also cell type-specific, indicating that GeneExt can rescue transcriptomic signal from previously undercounted cell types. This latter effect could be explained by different factors: (i) genes that are expressed in rare cell types could be absent in the bulk RNA-seq experiments commonly used for evidence-based gene annotation; or (ii) some specialized cell types dedicate a large fraction of their transcriptional output to one or a few genes (e.g. secretory or digestive cells producing proteases), and if these genes belong to families that are systematically mis-annotated for any reason (e.g. they are short or repetitive), this bias will have an outsized effect on these particular cells. Finally, we compared the performance of GeneExt with that of a similar tool: peaks2utr (Haese-Hill *et al.* 2023). To this end we artificially truncated gene model 3ʹ ends in three well-annotated model species (*D. melanogaster*, *C. elegans*, and *M. musculus*) and used available scRNA-seq datasets (Gupta *et al.* 2022, Li *et al.* 2022, Smith *et al.* 2024) to extend gene annotations with GeneExt and peaks2utr (Fig. S1). In small, gene-dense genomes, GeneExt showed superior accuracy in predicting the actual 3′ end of genes (Fig. S1a), and, consequently, more accurate gene expression quantification (Fig. S1b). Overall, these analyses demonstrate that GeneExt can ameliorate the often-unanticipated effects that gene annotation inaccuracies can have in the transcriptomes of particular species and cell types.

In summary, GeneExt is a versatile tool that refines existing gene annotations to improve scRNA-seq quantification across species. The software requires minimal input (a pre-existing annotation in any format and scRNA-seq reads) and can be used with default parameters suitable for most species. The result is improved gene detection that facilitates the interpretation of single-cell atlases. More broadly, standardizing gene detection and quantification across species is essential for comparative analyses of cell type gene expression, particularly as cell type atlases continue to expand taxonomically across the tree of life (Sebé-Pedrós *et al.* 2025).

## Supplementary Material

btag094_Supplementary_Data
