# Supplementary material for: GeneExt: a gene model extension tool for enhanced single-cell RNA-seq analysis
Source: Bioinformatics. 2026 Mar 2;42(3):btag094. doi: 10.1093/bioinformatics/btag094 (PMC12970594; doi:10.1093/bioinformatics/btag094)
Supplement: btag094_Supplementary_Data [file btag094_supplementary_data.pdf]

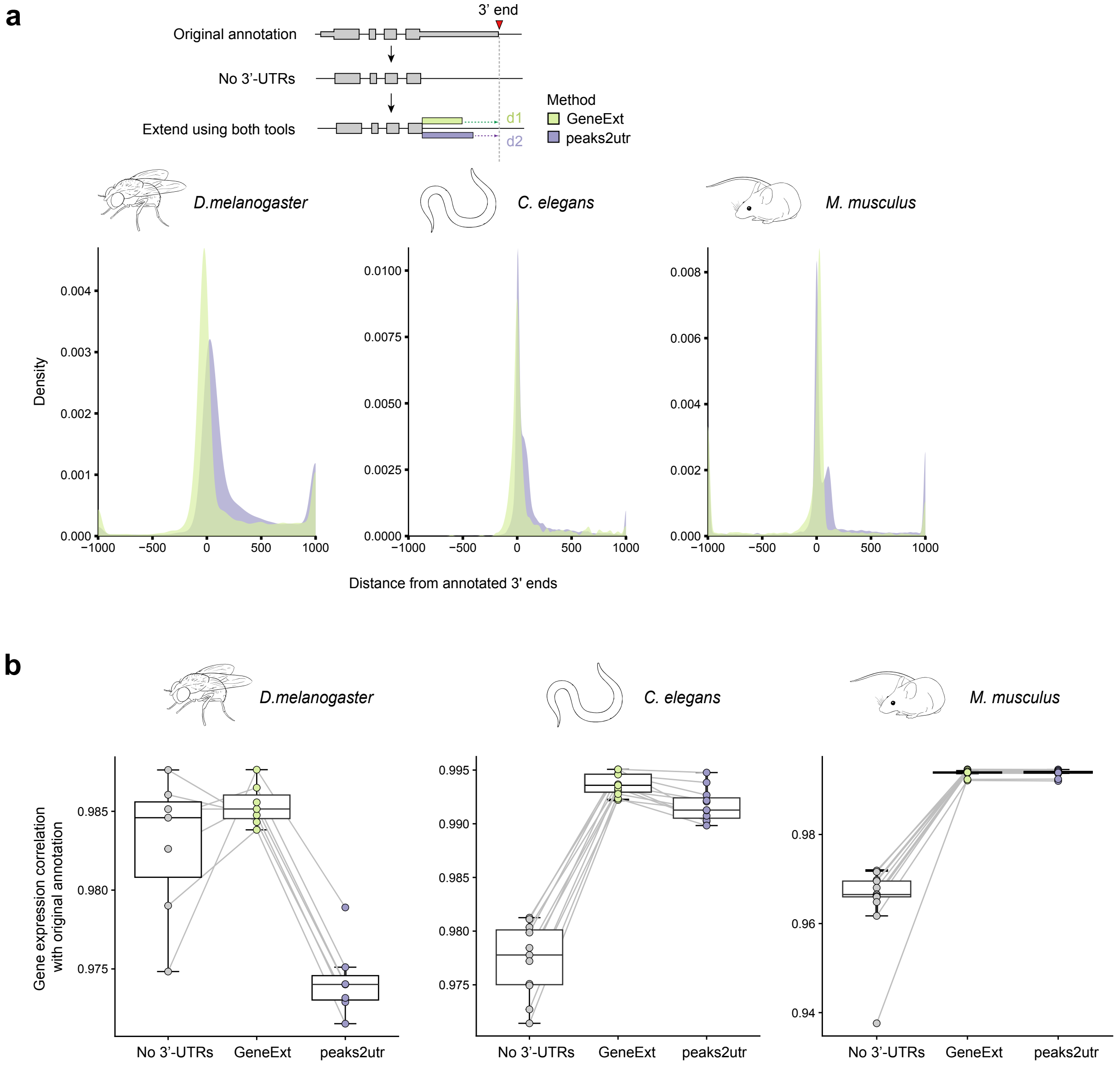

Figure S1. Benchmarking GeneExt in model organisms. a, Comparison of extended gene 3' ends to the original 3' ends in each of the species. The density plots show the distribution of distances (in bp) from the extended 3' ends to the original 3' ends. Distance values have been capped at -1000bp and 1000bp. b, Boxplot showing for each cell cluster the Pearson correlation between original gene expression values and gene expression values obtained with each of the modified gene annotations.
